# Prevalence, severity, and associated factors of depression in newly diagnosed people living with HIV in Kilimanjaro, Tanzania: a cross-sectional study

**DOI:** 10.1186/s12888-022-04496-9

**Published:** 2023-02-01

**Authors:** Kim Madundo, Brandon A. Knettel, Elizabeth Knippler, Jessie Mbwambo

**Affiliations:** 1 Department of Mental Health and Psychiatry, Kilimanjaro Christian Medical Centre, Moshi, Tanzania; 2 Duke University School of Nursing and Duke Global Health Institute, Durham, NC USA; 3 Duke Centre for AIDS Research, Duke University School of Nursing, Durham, NC USA; 4 Department of Psychiatry and Mental Health, Muhimbili University of Health and Allied Sciences, Dar es Salaam, Tanzania

**Keywords:** Depression, Mental health, Patient Health Questionnaire, HIV, Determinants of health, Newly diagnosed, Tanzania, Sub-saharan Africa

## Abstract

**Background:**

Depression is particularly common among people living with Human Immunodeficiency Virus (HIV), with some studies showing a prevalence of depression three times higher among people living with HIV as compared to the general public. The stress associated with being diagnosed with HIV can be quite impactful, including concerns about one’s long-term health, stigma, and the burden of long-term treatment. Therefore, it is common for a new HIV diagnosis to contribute to the onset of depressive symptoms. The objective of this study was to determine the prevalence and severity of depression, and its associated factors in people diagnosed with HIV within the past 12 months.

**Methods:**

We conducted a cross-sectional survey with patients newly diagnosed with HIV at three hospitals in the Kilimanjaro region of Tanzania utilizing a locally validated version of the Patient Health Questionnaire-9 (PHQ-9) as a screener for depression, the Demographic Health Survey (SES-DHS8) for socio-demographic characteristics, and the Duke-UNC Functional Social Support Questionnaire (FSSQ) to assess perceived social support. We enrolled 272 participants between September and December 2020, diagnosed with HIV within the past 12 months. Analysis of Co-variance (ANCOVA) and Bonferroni post-hoc analysis were used to determine associations of sociodemographic variables with the dependent variable of depression.

**Results:**

Overall prevalence of depression in our sample was 41%, including 54 participants (20%) with moderate symptoms, 42 (15%) with moderately severe symptoms, and 16 (6%) with severe symptoms. Severity was highest in participants diagnosed with HIV less than 1 month ago. An ANCOVA model (overall *F* = 4.72, *p* < 0.001) assessing factors associated with greater depression severity revealed significant effects of study site (*F* = 7.6, *p* < 0.001), female gender (*F* = 5.11, *p* = 0.02), and less time since HIV diagnosis (*F* = 12.3, *p* < 0.001).

**Conclusion:**

The study demonstrates very high prevalence of depression among people living with HIV in this setting, particularly among those newly diagnosed, female participants, and those seen at the larger regional referral hospital. Integration of mental health screening and interventions into CTC care is vital in the first visits following a positive test result and may be tailored to meet the needs of patients at highest risk for developing symptoms of depression.

## Background

There are approximately 38 million people living with HIV (PLHIV) worldwide [1], including 1.4 million in Tanzania, representing the fifth highest incidence of HIV of any nation [2]. To address the global HIV pandemic, research has focused on developing safe and effective preventive and therapeutic interventions [3]. Increasingly, treatment-related research is focusing on efforts to improve access and adherence to anti-retroviral therapy (ART), including addressing social determinants, mental health, and psychosocial barriers that may prevent ART initiation and long-term adherence [4].

Unfortunately, the stress associated with having a severe and chronic illness, as well as the neurological effects of HIV, have been shown to increase susceptibility to mental health challenges among PLHIV [5]. A bi-directional relationship has been noted between HIV/AIDS and mental disorders, with depression being the most common and most impactful [6].

The World Health Organization reports depression as one of the top three causes of burden of disease worldwide, and is expected to be the leading cause by 2030 [7]. Studies from across other continents [8–10], from Sub-Saharan Africa (SSA) [11, 12] and from within Tanzania [13, 14] show that depression can occur up to three times more frequently among PLHIV compared to those without HIV infection, with prevalence of depression being as high as 58% in some Tanzanian settings [15]. Symptoms of depression have been reported to be most severe in the period soon after HIV diagnosis [10, 16]. Depression remains largely underdiagnosed and undertreated [17], or even neglected [18], with reports of 40 to 85% with depression worldwide receiving insufficient intervention [17, 19]. Previous literature from other continents has identified female gender, age below 40, and being single or unmarried as associated factors of depression among PLHIV [20–22], while other studies from SSA have revealed a relationship between depression and level of education, social support, and employment [23–26]. Studies from SSA highlight depression as significantly associated with lower quality of life, longer recovery times, and higher rates of functional disability among PLHIV [27–29]. Poor HIV outcomes such as virological failure, drug resistance, and non-adherence to HIV treatment have been associated with depression in Tanzania and elsewhere [14, 15].

The event of diagnosing someone with HIV in itself can be considered a Stressful Life Event (SLE) [8]; causing an enduring emotional burden due to the knowledge of a serious and chronic illness, anticipated, internalized, or enacted stigma, and the burden of lifelong use of medications [30, 31], all of which may contribute to the onset of depression. In Tanzania, people newly diagnosed with HIV receive brief post-test counseling from an HIV-care nurse and may have access to a community health worker for adherence support. However, prior studies have demonstrated that these health workers have minimal formal training in mental health topics or counseling and face time and resource constraints that make it difficult for them to attend to the emotional needs of patients [32, 33]. Further, formal mental health treatment is limited in Tanzania and referrals for these services are rare [34]. As the development of such services becomes more of a priority in HIV care, it is critical to better understand the extent and nature of depression among people in HIV care.

To the best of our knowledge, no prior studies in Tanzania have systematically explored recent HIV diagnosis (within the past 12 months), prevalence and severity of depression, and its associated factors. In this study, we enrolled a sample of people newly diagnosed with HIV to investigate this. In light of findings from earlier studies [15, 35], we hypothesized that people with a newer HIV diagnosis, women, and those with lower social support would report greater symptoms of depression.

## Methods

This cross-sectional study was conducted at three purposively selected HIV Care and Treatment Clinics (CTCs) which are attached to hospitals in the Kilimanjaro Region of Tanzania: Mawenzi Regional Referral Hospital, Hai District Hospital and Majengo Health Centre. The health centres were chosen to include facilities of varying size, location (urban, suburban, and rural), and referral capacity with the intention to recruit a wider diversity of participants from first visit consultations (small health centre and district level) to those who had been referred due to more complex challenges (regional referral level).

Eligible patients were adults 18 years and above, diagnosed with HIV within the past 12 months, and able to provide informed consent. Participants who could not write their signature were able to document their informed consent with a fingerprint. Full informed consent forms were read aloud in the presence of an interpreter and a witness who also provided signatures in support of the participants’ informed consent. We excluded patients whose physical or mental condition made them unable to participate and those who had previously experienced a manic or hypomanic episode to rule out bipolar disorder. We ruled these conditions out by including two questions based on criteria A under the sections on manic and hypomanic episodes in the DSM 5. A ‘yes’ response to either of these questions led to exclusion from the study.

The desired sample size (*N* = 272) was calculated using an equation taken from Cochran’s formula: N=[Z^2^P(1-P)]/d^2^ whereby ‘N’ is the estimated desired sample size, ‘Z’ represents the confidence level at 95%, ‘P’ stands for the prevalence of depression, and ‘d’ represents the margin of error at 5% [36]. We used a previous study [35] from Tanzania to obtain an estimated prevalence of depression. We then recruited 272 individuals presenting for routine HIV care appointments at the three study sites. The number of participants recruited per study site was proportional to the number of patients seen at each clinic. For measures that were neither translated nor validated previously in Tanzania, a formal forward- and back-translation was done by two Muhimbili University of Health and Allied Sciences (MUHAS) staff members not part of the study team, then compared with the original tools for linguistic and cultural equivalence.

### Demographic information

We first administered a questionnaire to obtain socio-demographic information based on the Demographic Health Surveys Questionnaire (SES-DHS8 – Household Schedule) including variables such as age, gender, and level of education. This questionnaire has been used extensively in national studies in Tanzania [37, 38].

### Social support

We used the Duke-UNC Functional Social Support Questionnaire (FSSQ) for perceived level of social support. This instrument covers multiple dimensions of social support including emotional (e.g., “chances to talk about problems at work or with my housework”), physical/instrumental (e.g., “help when I am sick”) and social aspects (e.g., “invitations to go out and do things with other people”). The 14 items are rated on a Likert-type scale of 1 to 5, with higher scores representing higher social support and a maximum score of 70 [39]. For this study, scores were divided into quartiles with an inter-quartile range of 17.5 to create equally-weighted ordinal levels from poor (0-17.5), fair (17.6–35), good (35.1–52.5) and excellent social support (52.6–70). A recent study showed very good reliability, with a Cronbach’s alpha of 0.87 [40].

### Stressful life events

A screening checklist based on the Life Events Checklist for DSM 5 (LEC-5) tool was used to detect history of SLEs and identify other stressful or traumatic events that could be contributing to depressive symptoms, such as witnessing a sudden accidental death, and experiences of physical or sexual assault. The checklist was comprised of 16 specific items and an additional question for ‘others’ whereby responses are ‘Yes’ or ‘No’ [41]. It is based on similar tools with good test-retest reliability and internal consistency [42].

### Patient records

Official documentation was requested from the individual participants and CTC clinics to obtain details on the date of HIV diagnosis, the date of initiation of HIV treatment, and viral load and CD4 counts to corroborate information provided by the participant.

### Depression

The Patient Health Questionnaire 9 (PHQ-9) was utilized as a screening tool for depression. The tool has previously been translated to Kiswahili [43], and validated in the Tanzanian context, showing very good internal consistency (Cronbach’s alpha = 0.83) [44]. The PHQ-9 is comprised of 9 items which match the Diagnostic and Statistical Manual of Mental Disorders version 5 (DSM 5) criteria for major depressive disorder. These criteria are required to have been present for a minimum duration of two weeks [45], whereby each item can be scored from 0 (no symptoms at all) to 3 (symptoms nearly every day) with a maximum score of 27. The cut-off scores assigned were derived from the earlier validation study in Tanzania: 0–4 to indicate minimal severity, 5–9 (mild), 10–14 (moderate), 15–19 (moderately severe) and 20+ (severe symptoms) with a score greater than 9 being equivalent to a major depressive episode, or probable depression [44].

The surveys were piloted using five randomly selected participants at each study site to check for understandability. Data collection was performed by the principal investigator (PI) and two undergraduate research assistants who had prior experience in mental health and public health research. The research assistants received training in the content area and survey administration, and were required to demonstrate familiarity and competency with the survey in two full mock interviews with the PI prior to enrolling patients. Data collection was conducted in-person at the three study hospitals over a 4-month period from September to December 2020. The study was introduced to all patients visiting for routine care every morning in the reception area as the patients waited for their doctor’s visit. Eligible and interested participants were escorted to a private research office where they completed the informed consent procedure and the study survey.

Independent variables of interest were age, gender, employment status, level of education, perceived social support, distance from home to CTC, time from HIV diagnosis, and past SLEs. The dependent variable in this study was severity of depression. Participants were categorized according to age: 18–24, 25–49, and 50 or older; as male or female; according to distance from home to the respective CTC: <5kilmoetres, 5-10 km, or > 10 km; and according to the duration since HIV diagnosis: less than 1 month ago, between 1 to 3 months, between 3 to 6 months, or between 6 months and 1 year.

Continuous data were first summarized by calculating means, frequencies, standard deviations and ranges; categorical data were summarized through frequencies of responses.

Analysis of Co-variance (ANCOVA) was used to determine the associations between categorical and continuous independent variables and the dependent variable of depression. For variables significant in the initial multivariable model, additional testing was conducted using Bonferroni post-hoc analysis.

All study procedures were performed in accordance with the Declaration of Helsinki regulations.

## Results

Among the 272 participants, the mean age was 41 (SD ±12), with a range of 18 to 75 years. 61% of the participants were female and the majority (68%) had a primary school education. More than one-third (42%) of participants were living with family, one-third (33%) were living alone, and 24% were living with a spouse or partner. More than one-third (42%) of participants lived further than 10 km from the respective CTC. Sixty-two (23%) of the participants had been diagnosed with HIV less than month prior to the interview, 43 (16%) were diagnosed between 1 and 3 months prior, 52 (19%) between 3 and 6 months prior, and 115 (42%) between 6 and 12 months prior to the interview. More than half (56%) of the participants reported an excellent level of perceived social support, although the sample mean (52.2) fell in the ‘good’ category.

**Table 1:** Sample characteristics (*N* = 272)

Variable	Observations n (%)
**Study site**	
Mawenzi Regional Referral Hospital	114 (41.91)
Hai District Hospital	98 (36.03)
Majengo Health Centre	60 (22.06)
**Age (mean = 41)**	
18–24	24 (8.82)
25–49	184 (67.65)
50+	64 (23.53)
**Gender**	
Male	106 (38.97)
Female	166 (61.03)
**Level of education**	
None or informal	21 (7.72)
Primary education	185 (68.01)
Secondary education	49 (18.01)
Higher education	17 (6.25)
**Living situation**	
Alone	89 (32.72)
Spouse or partner	65 (23.90)
Family or relatives	115 (42.28)
Friend	2 (0.74)
Other	1 (0.37)
**Type of employment**	
Unemployed	59 (21.69)
Throughout the year	135 (49.63)
Seasonal	78 (28.68)
**Distance from home to CTC**	
< 5 km	85 (31.25)
5–10 km	73 (26.84)
> 10 km	114 (41.91)
**Duration since HIV diagnosis**	
< 1 month	62 (22.79)
1–3 months	43 (15.81)
3–6 months	52 (19.12)
6–12 months	115 (42.28)
**Perceived level of social support**	
Poor (0–17.5)	1 (0.37)
Fair (17.6–35)	38 (13.97)
Good (35.1–52.5)	87 (31.99)
Excellent (52.6–70)	146 (56.38)

The prevalence of depression in this sample was 41% using a cut-off score above 9 indicating likelihood of moderate to severe forms of depression (Table 1). In terms of perceived difficulty of functioning due to the depression, more than half of participants (57%) reported difficulty in areas such as occupation, academics, and social interactions (Table 2).

**Table 2:** Prevalence of depression and perceived level of difficulty in functioning over the past two weeks (*N* = 272)

Variable	Outcome	n (%)
**Depression**	**Severity**	
	None/Minimal (PHQ 0–4)	81 (29.78)
	Mild (PHQ 5–9)	79 (29.04)
	Moderate (PHQ 10–14)	54 (19.85)
	Moderately severe (PHQ 15–19)	42 (15.44)
	Severe (PHQ 20+)	16 (5.88)
**Level of difficulty to function**	Not difficult at all	117 (43.01)
	Somewhat difficult	96 (35.29)
	Very difficult	38 (13.97)
	Extremely difficult	21 (7.72)

**Fig. 1:**
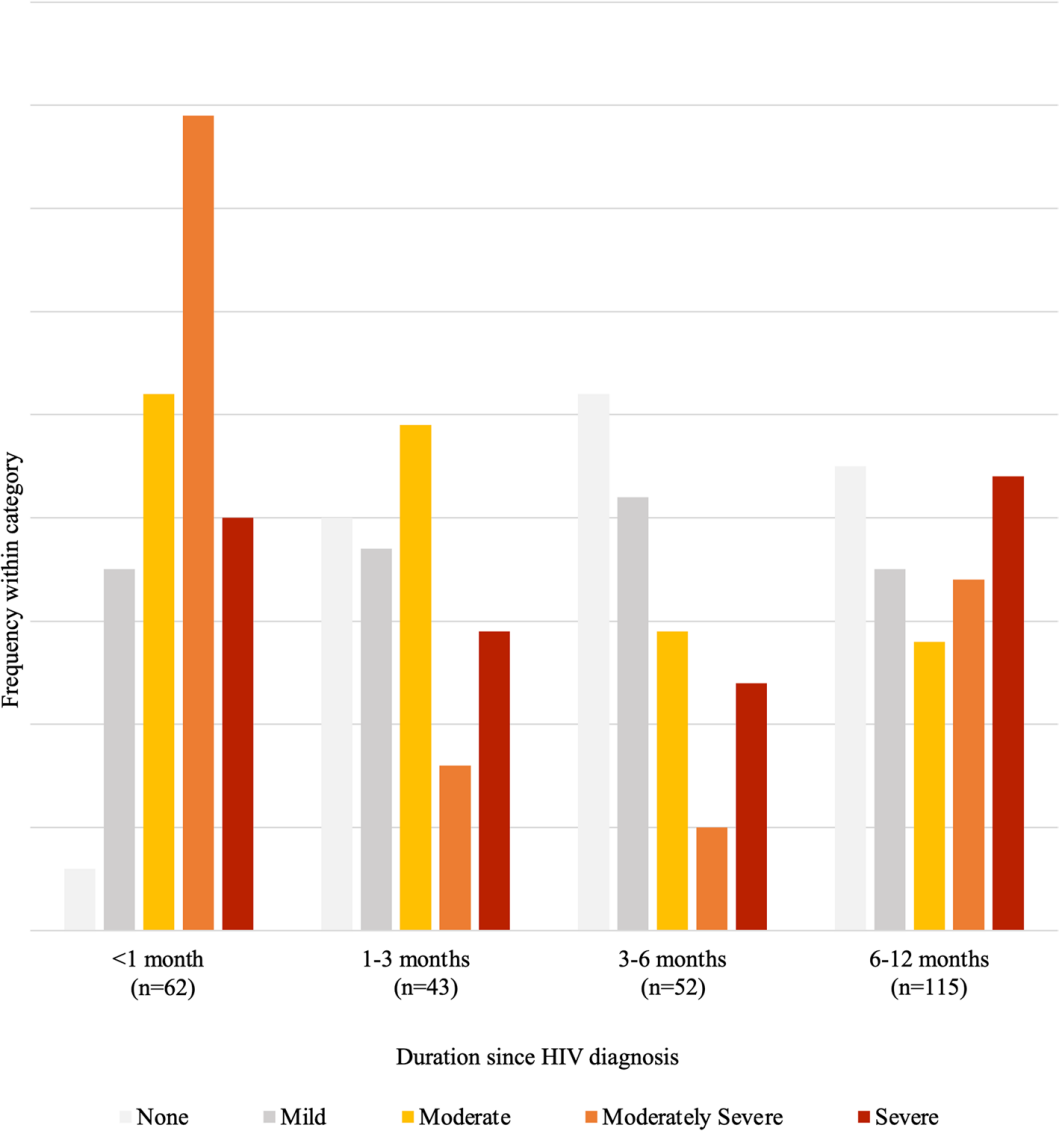
Severity of depression by category of time from HIV diagnosis (*N* = 272)

The graph above (Fig. 1) shows that of the 62 participants diagnosed with HIV less than 1 month before the survey, there was a higher severity of depressive symptoms (also see Table 4). The mean PHQ-9 score was highest in this recently diagnosed group (13±5) and lower in the other groups of participants (time from HIV diagnosis: 1–3 months: [8±6], 3–6 months: [7±6], and 6–12 months: *n* = [8±7]).

An ANCOVA was run on the data for 272 participants to examine the effect of nine independent variables on a single dependent variable (total PHQ-9 score). The ANCOVA model (overall F-ratio = 4.27, *p* < 0.001) revealed significant effects of study site, age and duration since HIV diagnosis on depression severity (Table 3).

**Table 3:** Associations of independent variables with depression severity as measured by PHQ-9 (*n* = 272)

Variable	df	F	*p*
Study site	2	**7.55**	**< 0.001**
Age	1	0.37	0.69
Gender	1	**5.11**	**0.02**
Level of education	3	2.08	0.10
Living arrangement	4	2.23	0.07
Type of employment	2	1.04	0.35
Duration since HIV diagnosis	3	**13.04**	**< 0.001**
Distance from home to HIV clinic	2	0.37	0.69
Perceived level of social support	3	2.48	0.06
**Overall**	22	**4.27**	**< 0.001**

R-squared = 0.27, Adj. R-squared = 0.21

Bonferroni post-hoc analysis was conducted for the three significant independent variables (Table 4). Patients in the ‘<1 month’ group showed a significantly greater severity of depressive symptoms than in all of the other groups (all *p* < 0.001). Participants interviewed at Mawenzi Hospital, the large regional referral site, also reported greater severity of depressive symptoms compared to Majengo Health Centre, a smaller urban hospital (*p* = 0.009), and Hai district hospital, a small rural hospital (*p* = 0.01). Female participants had a significantly higher mean depressive symptom score (9.3±6.3) than men (8.3±6.3) (*p* = 0.02).

More than two-thirds (*n* = 189, 69.5%) of participants responded ‘Yes’ to having experienced a past SLE based on the 16-item checklist; the most common SLE was involvement in a serious transportation accident (*n* = 37, 13.6%) and the least common being held captive (*n* = 1, 0.37%). Given the low frequencies of many SLE, these were not included in the ANCOVA model. Chi-square was used to analyse for associations between SLE and probable depression. Significant relationships were only detected among three – severe suffering (*n* = 8, *p* = 0.002), witnessing a sudden violent death (*n* = 7, *p* = 0.01), and serious injury, harm or death you caused to someone else (*n* = 2, *p* = 0.03). The SLEs not showing significant association with depression include: natural disaster (*n* = 15, *p* = 0.33), fire or explosion (*n* = 7, *p* = 0.79), transportation accident (*n* = 37, *p* = 0.95), serious accident at work or home (*n* = 5, *p* = 0.54), contact with a toxic substance or radiation (*n* = 7, *p* = 0.23), physical assault (*n* = 33, *p* = 0.48), assault with a weapon (*n* = 16, *p* = 0.21), sexual assault (*n* = 14, *p* = 0.3), other unwanted or uncomfortable sexual experience (*n* = 8, *p* = 0.09), combat or exposure to a war-zone (*n* = 2, *p* = 0.84), being held captive (*n* = 1, *p* = 0.24), life-threatening illness or injury (*n* = 3, *p* = 0.74), and witnessing a sudden accidental death (*n* = 24, *p* = 0.68).

**Table 4:** Bonferroni post-hoc comparisons of independent variables with significant effect on participant depressive symptoms

Variable	Study site		
	Mawenzi		Majengo
Majengo	*F*=-2.97		
	***p = 0.009***		
Hai	*F*=-2.43		*F* = 0.54
	***p = 0.01***		*p = 1*
	**Duration since HIV diagnosis**		
	**< 1 month**	**1–3 months**	**3–6 months**
1–3 months	*F*=-4.78		
	***p*** **< 0.001**		
3–6 months	*F*=-5.63	*F*=-0.86	
	***p*** **< 0.001**	*p* = 1	
6–12 months	*F*=-4.25	*F* = 0.53	*F* = 1.39
	***p*** **< 0.001**	*p* = 1	*p = 1*

## Discussion

Depression is the most commonly occurring mental health challenge related to HIV, and contributes to substantially higher burden of disease among PLHIV. This study aimed to estimate the prevalence of depression and to determine factors associated with depression among newly diagnosed PLHIV attending CTCs in Kilimanjaro, Tanzania.

The prevalence of depression in this sample was 41.18% using a PHQ-9 cut-off score indicating moderate to severe forms of illness; these findings are comparable to data from previous studies in similar populations from different parts of the world and substantially higher than the prevalence observed in the general public [8, 10], highlighting the magnitude of mental health challenges among PLHIV. Studies from Tanzania have also shown high prevalence of depressive symptoms among PLHIV ranging from 23 to 58%, though the use of different screening tools [15], varied sub-populations of focus (such as exclusively pregnant women) [46] and different types of study sites [13] make direct comparison difficult.

The high prevalence of depression detected in this study indicates there is a pressing need for integration of screening and interventions into CTC care for improved detection and early management of mental health issues such as depression. There are strong examples of evidence-based mental health interventions in the context of HIV care. For example, the mhGAP (Mental Health Gap Action Programme) created by the World Health Organization in 2008 was purposely designed for integration of screening, diagnosis, basic intervention, and appropriate referrals for people with common mental disorders (such as depression) at the primary health care level in low- and middle-income countries [47]. A task-shifting intervention known as the Friendship Bench has been used by training lay counsellors in primary care clinics in Zimbabwe to identify and treat people with moderate to severe depression [48], even proving effective in populations of PLHIV [49]. Such training models and implementation could be extended to CTC care in Tanzania, even where there is a shortage of mental health professionals.

Additionally, the Tanzania National Guidelines for the Management of HIV/AIDS contains a section on mental disorders associated with HIV and highlights the necessity of timely initiation of treatment for depression including low-dose antidepressants and referral to mental health services [50]. These guidelines could be upgraded to recommend routine screening and brief intervention for depression such as psychoeducation be done in the visits following HIV diagnosis as well as include summarized standard operating procedures on linkage to specialized care. In preliminary qualitative studies, both nurses and community health workers in HIV care in Tanzania have expressed interest in more structured counseling training [51, 52].

This study reveals that the severity of depression was highest in participants seen less than 1-month post-HIV diagnosis. Similar findings have been reported in studies from Sub-Saharan Africa [16, 53] and China [8]. These studies were conducted in similar settings to our current study; all outpatient clinics, and the former two in rural and semi-urban clinics. They all emphasized the need for appropriate intervention to address the depressive symptoms starting immediately upon diagnosis and continuing during early follow up visits.

The severity of depression being highest in the first month after HIV diagnosis is suggestive of the prominent and stressful nature of the event of being diagnosed with HIV. The deeply sensitive nature of being diagnosed with a severe and chronic illness, the accompanying information on the necessary life-long use of medications, anticipation of stigma, concerns regarding disclosure to others, and resulting apprehension about social support all add weight to the probability of HIV diagnosis as a contributing factor to the onset of depressive symptoms or exacerbation of existing symptoms [8, 12]. If testing was prompted by the onset of HIV or AIDS-related symptoms, the patient may also be facing considerable mental health concerns at the time of diagnosis [54].

Given the likelihood of depression at such a critical time in the treatment of HIV, it is essential that providers screen for depressive symptoms and educate newly diagnosed PLHIV about the risks of depression in a non-stigmatizing way, including how to recognize it and the steps to take in seeking help if symptoms arise. The mhGAP Programme is an example of a package that could guide clinical practitioners in CTC care on pharmacological and basic psychosocial interventions such as psychoeducation and problem-solving therapy, targeted towards clinically significant depression [47, 48].

We also observed higher severity of depression at the larger regional referral hospital. A study from Cameroon similarly reported high prevalence (63%) of depression at referral level centres [12]. Given its role in the health system and higher number of specialized staff, this hospital often receives referrals of clients with more severe illness that could not be appropriately managed at lower-level centres. Unfortunately, mental health services at this hospital are disproportionately underfunded and understaffed, which limits the capacity to respond to all but the most severe mental health challenges [55]. Strengthening mental health services at all levels of care and reinforcing referral pathways in the region could potentially address this. Additionally, we believe that selecting study sites of varying size, location, and at different levels in the referral system added conceptual value to our study since it allowed for this distinction.

ANCOVA findings also revealed a significant effect of female gender on depression, which has been highlighted in other studies from around the world [56, 57], SSA, and Tanzania [23]. Women have better health-seeking behaviour than their male counterparts [15], which could lead to higher detection rates of depression among women. However, women are also often more vulnerable to traumatic events than men, and experience higher rates of internalized stigma and caregiving stress that could also contribute to depressive symptoms [58–60].

Remarkably, level of education was not significantly associated with depression. While previous literature has highlighted this association, an explanation for this difference in our findings could be that having no education, or incomplete primary education is more likely associated with depression [61]. In this study, more than 92% of the participants had completed at least primary level of education. Similarly, there was no association between perceived level of social support and depression, which is contrary to recent studies from SSA indicating social support as a key determinant of depression [15, 24]. A possible explanation for this is that majority of participants (89%) had either good or excellent perceived level of social support, and only one participant identified themselves as having poor social support.

Other factors such as age, marital status, and type of employment were not significantly associated with depression. Prior studies have supported each of these variables as potential predictors of depressive symptoms among people living with HIV in African settings [15, 23, 62–66]; however, population-level and methodological differences may account for the disparate findings in the current study.

Among the 16 SLE screened through a checklist, three were revealed to have significant associations with depression, suggesting a potential syndemic relationship between SLEs, depression, and living with HIV. The three that were associated with depression - severe suffering, witnessing a sudden violent death, and causing serious harm or death to someone else – could be hypothesized to be among the most serious or traumatic SLE among the 16, which might support earlier research that a highly traumatic event can have serious long-term impacts on depressive symptoms in the context of chronic disease [67]. Future research may seek to enroll a greater number of PLHIV who have experienced traumatic stress to examine implications for mental health and HIV care.

### Limitations

Depression and HIV are both highly stigmatized conditions; therefore, there is a risk of social desirability bias in this self-report study. Throughout the research process, research staff reassured participants of the confidentiality of their responses and were trained to respond compassionately to topics with high potential for stigma. The choice of interviewer-administered assessments (as opposed to self-administration via questionnaire) may have increased the likelihood of social desirability bias; however, based on our past experience in this setting, we felt the ability for the interviewer to manage data quality in real time outweighed this concern. Due to the cross-sectional nature of this study, it is not possible to determine direction or causality among the independent and dependent variables and we have interpreted the data accordingly. It is also not possible to determine from these data how depressive symptoms may have changed over time. Finally, a diagnosis of depression is preferably made through a clinical interview provided by a mental health professional. Although we used a version of the PHQ-9 validated in Tanzania such a measure is not an ideal proxy for the gold standard clinical assessment. Future studies may seek to corroborate PHQ-9 validity in the HIV setting in the Kilimanjaro region.

## Conclusion

We examined the prevalence, severity and associated factors of depression among newly diagnosed PLHIV in Kilimanjaro, Tanzania. Prevalence of depression was high in this setting. Being newly diagnosed with HIV, attending a larger referral hospital, and being female were associated with greater depressive symptoms. It is important to note that depressive symptoms have been associated with a negative impact on HIV treatment adherence and engagement with CTC care, especially in the initial period of treatment. Adding to previous literature, the high prevalence of depression detected in this study indicates that depression can occur very early during HIV treatment. Integration of screening and interventions into CTC care is pertinent, particularly in the early days after an HIV diagnosis. Early detection can provide an opportunity to begin timely mental health intervention or referrals between CTC centres and mental health facilities for further care. Furthermore, we recommend that newly diagnosed PLHIV should be made aware of the risk of developing depression soon after HIV diagnosis, how to recognize it, and the steps to take in seeking help if symptoms arise.

## Data Availability

Deidentified datasets used and/or analysed during the current study are available through URL from the corresponding author on reasonable request.

## Abbreviations

AIDS: Acquired Immunodeficiency Syndrome
ANCOVA: Analysis of Co-Variance
ART: Anti-retroviral Therapy
CTC: Care and Treatment Clinic
HIV: Human Immunodeficiency Virus
PHQ-9: Patient Health Questionnaire-9
PLHIV: People Living with HIV
SLE: Stressful Life Event
